# Building and sustaining infection prevention and control teams at two pediatric cancer units in Ecuador and Guatemala through a collaboration partnership

**DOI:** 10.3389/fonc.2025.1577066

**Published:** 2025-09-25

**Authors:** Joanna J. Acebo, Mario A. Melgar, Maysam R. Homsi, Miriam L. Gonzalez, Alicia C. Cojulun, Federico Antillon-Klussmann, Jose M. Eguiguren, Elaine I. Tuomanen, Miguela A. Caniza

**Affiliations:** ^1^ Pediatric Oncology Service, Hospital SOLCA Quito, Quito, Ecuador; ^2^ Unidad Nacional de Oncología Pediátrica, and School of Medicine, Francisco Marroquin University, Guatemala, Guatemala; ^3^ Department of Global Pediatric Medicine, St. Jude Children’s Research Hospital, Memphis, TN, United States; ^4^ Hospital Metropolitano de Quito, Quito, Ecuador; ^5^ Department of Host–Microbe Interactions, St. Jude Children’s Research Hospital, Memphis, TN, United States; ^6^ Department of Infectious Diseases, St. Jude Children’s Research Hospital, Memphis, TN, United States; ^7^ Department of Pediatrics, University of Tennessee Health Science Center, Memphis, TN, United States

**Keywords:** pediatric, cancer, low- and middle-income countries, infection prevention and control, link teams, Ecuador, Guatemala

## Abstract

**Background:**

Hospital infection prevention and control (IPC) programs are often insufficient to meet the needs of pediatric oncology units (POUs) in low-resource settings. Accordingly, we established partnerships to build and sustain dedicated IPC teams for two POUs in Ecuador and Guatemala.

**Methods:**

Each partnership comprised four phases: (1) planning and preparation; (2) developing the IPC team; (3) sustaining the IPC team; and (4) integrating the IPC team into the institution. The impact of the IPC teams was assessed by monitoring healthcare-associated infections (HAIs) and compliance with IPC practices.

**Results:**

At Hospital SOLCA–Quito, Ecuador, local champions were identified and trained. These in turn built local IPC teams that led healthcare improvement by using surveillance for outcome measures, monitoring practices for processes measures, and staff training. As the collaboration progressed, infection rates decreased steadily. At SOLCA–Quito, there were 9 HAIs/1000 patient days at baseline in 2010, whereas at the end of 2019, there were 2.6 HAIs/1000 patient days. A similar program was developed at the UNOP hospital in Guatemala, where the HAI rate decreased from 9.9/1000 patient days in 2011 to 5.37/1000 patient days in 2019 and the CLABSI rate decreased from 32.75/1000 catheter days in 2008 to 3.11/1000 catheter days in 2019. Towards the end of the collaborations, the IPC teams were integrated into the institutional structures. The Ecuadorean IPC team was integrated as a link team between the pediatric oncology service and the hospital IPC program. The Guatemalan team became the institutional IPC program staff.

**Conclusions:**

Our collaborations decreased HAIs in two POUs. The model proved sustainable and became part of the institutional structures, and it has been replicated in POUs elsewhere.

## 1 Introduction

Healthcare-associated infections (HAIs) are common adverse events in hospitalized children (1, 2).

In low- and middle-income countries (LMICs), the proportion of hospitalized children who develop HAIs may be as high as 18% in critical care units (3). HAIs affect the morbidity, mortality, and quality of life of patients, and they significantly increase costs (4). Accordingly, healthcare facilities, national programs, and international organizations are focusing on this problem (5).

In pediatric patients with cancer, HAIs carry additional risks because of the underlying disease, treatment-related cytopenias (6), and the use of vascular access devices (6). Zermatten et al. reported 703 episodes of fever in 291 children during periods of neutropenia (7), and the reported rates of serious infection, including bacteremia, have ranged from 12% to near 30% (8–11). Timely management of these infections improved survival, but admissions to the ICU were more frequent when antibiotic administration was delayed (12, 13). Therefore, every effort must be directed towards optimizing the quality of healthcare delivery for this vulnerable population, including preventing HAIs.

The St. Jude Global Infectious Disease Program (GIDP) (formerly Infectious Disease International Outreach) was created in the early 2000s to support healthcare providers in pediatric oncology units (POUs) at global sites, especially in low-income settings, and to increase their infection care and infection prevention and control (IPC) expertise. Since its inception, the St. Jude GIDP has focused on IPC training and on mentoring healthcare providers, mainly—but not exclusively—in POUs. Many of these trainees have improved the infection care and IPC in their POUs and have strengthened the relationship with their institutional IPC programs. In selected situations, the St. Jude GIDP has equipped POUs with essential supplies. These supplies have included—but have not been limited to—hand hygiene soaps, antiseptics for vascular access, vascular catheters, ultrasound systems to locate veins, blood culture bottles, microbiology laboratory supplies, and educational materials relating to infection care and IPC. In this report, we assess the effectiveness of a collaborative effort to establish and support dedicated infection care and IPC teams for POUs in Ecuador and Guatemala.

## 2 Materials and methods

### 2.1 Settings

The two participating POUs were at hospitals in Ecuador and Guatemala. In Ecuador, the Hospital Oncológico SOLCA “Solón Espinosa Ayala” in Quito (SOLCA–Quito) is one of 10 network hospitals of the Sociedad de Lucha Contra el Cáncer (SOLCA). It is a 160-bed hospital for adult and pediatric patients, with 40 beds being dedicated to children with cancer. SOLCA–Quito opened in 1951 and has 900 full-time employees. In 2007, the IPC program of SOLCA–Quito had just one infection preventionist (IP), who worked with a multidisciplinary committee, and the functions of the IPC program were limited to passive, spotty surveillance, providing inconsistent reports of institutional IPC program performance to the hospital administration, and conducting minimal staff training in IPC. The staff of the SOLCA–Quito POU consisted of one oncologist and rotating general medicine residents. Because of the increasing number of pediatric patients receiving care in the POU and the frequent infections, many of them fatal, the medical personnel were expanded by hiring two dedicated pediatricians. In 2007, the SOLCA–Quito POU team reached out to the St. Jude GIDP, and the two entities agreed to collaborate to improve the local expertise in, and performance of, infection care and IPC in the POU.

In Guatemala City, the Unidad Nacional de Oncología Pediátrica (UNOP) is an 82-bed pediatric hospital for children with cancer that absorbs 65% of all pediatric cancer cases in Guatemala. UNOP opened in 2000 and currently cares for more than 500 new patients with cancer each year. UNOP has 107 administrative personnel, 306 nurses, 23 pediatricians, 13 oncologists, 12 oncology fellows, five intensivists, two critical care fellows, and three infectious disease (ID) specialists (two part-time and one full-time). The IP staff includes two full-time IPs and a nurse for data collection. In 2008, at the start of the St. Jude GIDP collaboration partnership, UNOP had 37 beds, with one nurse for IPC and a part-time infectious diseases consultant who chaired the IPC committee. Like their counterparts at SOLCA–Quito, the IPC program members had received suboptimal training, and their performance required improvement. The St. Jude GIDP agreed to collaborate with UNOP to improve the IPC performance and infection care management there.

### 2.2 IPC collaboration partnerships

During the IPC collaboration partnership, efforts were focused on multiple actions distributed across four main phases, namely, planning and preparing the intervention, building and nurturing the IPC team, sustaining the IPC team, and assimilating the IPC team (**Table 1**).

**Table 1:** Actions and resources for each phase of the IPC team-building period.

Phases	Actions (Responsible Party)
Planning and preparation	Local hospital and POU IPC evaluation (St. Jude GIDP site visit team)
	Resource procurement (St. Jude GIDP)
Developing the IPC team	Training local team members (St. Jude GIDP)
	Training material preparation: HH training; VA training—PICCs (St. Jude GIDP)
	Mentoring local team to obtain data and provide IPC reports (St. Jude GIDP)
	Data collection form preparation and database construction (St. Jude GIDP)
	Establishing formats for reports (St. Jude GIDP)
	Establishing infection definitions (St. Jude GIDP)
	Obtaining HH evaluation tools (St. Jude GIDP)
Sustaining the IPC team	Fine-tuning of team performance (St. Jude GIDP and local IPC team)
	Presenting team performance results (local IPC team and St. Jude GIDP)
Integrating the IPC team into the institution	Obtaining local institution support for teams and activities (local IPC team)
	Incorporating the IPC team and its functions into the institutional structure and operations (local IPC team)

GIPD, Global Infectious Disease Program; HH, hand hygiene; IPC, infection prevention and control; PICCs, peripherally inserted central catheters; POU, pediatric oncology unit; VA, vascular access.

During the first phase, planning and preparation, St. Jude GIDP members visited Ecuador and Guatemala; assessed the hospitals, the POUs, and the existing IPC resources; and engaged with the local institutional leadership regarding infection care and IPC and aligned with their goals. Also, during this first phase, the St. Jude GIDP obtained financial support for the collaborations.

In the second phase, developing the IPC team, the IPC team members were identified and trained; IPC data collection forms were prepared and a database was constructed using Epi Info [a CDC free software (14)]; patient data were protected, with only anonymized data being used for analysis; the IPC monthly report items were selected; and meeting formats were established. Importantly, the respective roles of the St. Jude GIDP members and the local IPC teams were delineated. During this phase, the local IPC teams began to perform their duties.

In the third phase, sustaining the IPC team, efforts were directed towards refining the quality of the local IPC teams’ performance with regard to infection surveillance; auditing selected IPC practices (hand hygiene, blood cultures, peripheral intravenous [PIV] line use, and phlebitis events); reporting outcome and process metrics (with adherence to established definitions and best practice guidelines); arranging healthcare provider participation in IPC trainings (on hand hygiene, safe vascular access, obtaining blood cultures, and isolation precautions); and sharing the reports of IPC data analysis results within the institutions and at meetings in the form of oral presentations or abstracts.

In the fourth and final phase, integrating the IPC team into the institution, efforts were directed towards encouraging the local leadership to accept the support of the created resource (the IPC teams) and to integrate the IPC team activities into the regular institutional IPC programs.

POU leaders at SOLCA–Quito and UNOP agreed to collaborate with St. Jude to improve IPC efforts in their POUs. The agreement facilitated the establishment of an IPC team and the identification and establishment of the team functions, and it provided support for implementing IPC processes (surveillance, hand hygiene, vascular access, and blood culture), which were planned and implemented in a stepwise fashion.

During scheduled monthly reporting sessions, conducted via a virtual platform, the St. Jude GIDP members and the SOLCA–Quito and UNOP IPC teams discussed issues relevant to the local teams, the POUs, and the respective healthcare institutions and provided their reports in the established formats. The reports consisted of a monthly IPC team performance assessment and a review and discussion of the outcome and process metrics. The data were anonymized and contained no patient identification. These metrics included the HAI rates, the infection present on admission (IPA) rates, the central line–associated bloodstream infection (CLABSI) rates, the rates and severity of phlebitis, the catheter-associated urinary tract infection (CAUTI) rates, and the ventilator-associated pneumonia (VAP) rates. The teams also reported on the total positive blood cultures, the percentage of positive blood cultures, the total positive cultures other than blood cultures, the types of pathogens isolated in blood cultures, the types of pathogens isolated in positive cultures other than blood cultures, and the susceptibilities of the pathogens. In addition, the teams reported the process metrics, including healthcare personnel compliance with hand hygiene, the total amount of alcohol gel used, and the number of healthcare providers and family members trained in hand hygiene and in precautions against respiratory infections (respiratory etiquette). As part of the collaboration partnership, the GIDP provided the local physicians with a supplemental salary, a laptop computer, essential reference books, membership in the Association for Professionals in Infection Control and Epidemiology (APIC), and financial support to attend selected annual medical specialty conferences during the collaboration. Throughout the partnership period, the local IPC team was encouraged to provide their hospital leaders and their institutional IPC program with the same reports as used in the virtual monthly reports to the GIDP and to gradually integrate the oncology IPC team functions into the institutional IPC program and other quality initiatives at their institution.

The hospital quality committee and the medical management of SOLCA–Quito and the academic committee of UNOP absolved the Ethics Committee from approving the study because it was determined that the information presented in the report did not constitute human subject research as all of the data were anonymized and the study did not use human subjects.

## 3 Results

### 3.1 Collaborative phases

Planning and preparation: During the first phase (2007–2009), we obtained local buy-in to the collaboration project by key stakeholders and local leaders, identified our local champion (15, 16), and obtained training, financial, and technical support for infection care and IPC in the POU.

Developing the IPC team: During the second phase (2010–2012), the following activities were undertaken: identifying local team members, formulating training and job performance descriptions, conducting needs assessments for infection care and IPC in the POU, and creating and obtaining approval of IPC policies and procedures. The local teams used available IPC technical information and guidelines from various sources, including the APIC Text (17), the Ecuador Ministry of Health (18), the Guatemala Ministry of Health (19), St. Jude IPC policies and procedures (20), the IPC policies and procedures of the Instituto Nacional de Pediatría in Mexico City (21), and the Mexican Health Secretariat (22). The local teams audited and provided feedback on IPC practices (surveillance of HAIs, hand hygiene, safe vascular access, surgical site infection, and urinary catheter usage) during the monthly meetings and also provided a summary of the monthly reports to their local oncology leaders.

Sustaining the IPC team: During the third phase (2013–2019), the surveillance, training, and auditing of IPC practices by the local IPC team became more prominent. Local facility leaders depended on the team for information on infection rates, hospital staff training, and interventions to improve the quality of care. The team members participated in meetings, they were consulted on matters pertaining to IPC, and their recommendations were respected. At SOLCA–Quito, the IPC team continued to work in the POU, but they collaborated closely with the institutional IPC program, whereas at UNOP, the IPC team members became the staff of the local IPC program.

Integrating the IPC team into the institution: During the fourth phase (2019–2023), the salaries of IPC team members were absorbed by their institution, the policies and procedures developed by the IPC teams were incorporated into the institutional policies and the normative standard, and the functions of the IPC teams were incorporated into the routine operations of the institution. The IPC interventions regarding surveillance, training, and auditing were adopted by the institutions. The monthly reports became part of the UNOP IPC reports, the SOLCA–Quito reports were used by the institutional IPC program, and the pediatric ward policies and procedures and the functions of the IPC team became institutionalized.

### 3.2 Building the local IPC team

At SOLCA–Quito and UNOP, a local pediatrician who was willing to work in infection care and IPC in the POU and willing to engage with the St. Jude GIDP was identified as a local champion (15, 16). The pediatricians (J.J.A. at SOLCA–Quito and M.M. at UNOP) completed 2 years of pediatric ID fellowship training at hospitals in Mexico City: one (J.J.A.) at the Instituto Nacional de Pediatría and the other (M.M.) at the Hospital Infantil de México. Thereafter, they participated in a series of focused trainings offered by the GIDP (23, 24) to enhance knowledge of, and expertise in, infection and IPC in immunocompromised children and to equip local pediatric ID specialists with the skills required to build and manage a local IPC team. Each of the training courses available to IPC team members used a combination of virtual and in-person training and assessed the satisfaction, knowledge, and skill acquisition of the participants. A certificate of completion was awarded at the end of the training. Importantly, there was continuous mentoring during the virtual monthly report meetings, with a “just-in-time” learning strategy (25) being used to build and sustain the team expertise in IPC throughout the collaboration period. Other local IPC team members at each site included a nurse IP and a data manager. The nurse IPs were trained using the GIDP training course (24). At SOLCA–Quito, throughout the collaboration period, the data manager role was shared by rotating pediatric residents and other IPC team members. At UNOP, a full-time dedicated data manager for data entry and secretarial activities was appointed. Data managers were trained in using Epi Info as a database. The IPC teams at SOLCA–Quito and UNOP had excellent working relations with other healthcare providers. For example, at SOLCA–Quito, surgical personnel were an integral part of the IPC team effort; they collaborated in following the best practice in placing central lines (26) and in measures to prevent surgical infections (27). At UNOP, the microbiologists were an integral part of the team. They participated in training and educational events promoted by the GIDP.

### 3.3 Performance metrics

The performance of the infection care and IPC teams was reflected in the results of infection surveillance of children admitted to the in-patient wards of the POU and in the adherence to hand hygiene, vascular access, and blood culture good practices by the healthcare workers.

- Surveillance of infections and their risk factors was accomplished by filling out a data collection sheet to report each admitted patient, any infections and risks of infections. We called this the “Blue Sheet” because the printed sheet was light blue in color (see **Supplementary Materials**). The Blue Sheet captured patient data in five categories: demographics, exposure to risk factors, infections diagnosed at or during admission, antimicrobials used, and susceptibilities of any microorganisms isolated. Data quality was managed by procedures followed by the GIDP and the UNOP/SOLCA– Quito teams. These included using a manual of procedures and HAI definitions; training in data collection, data processing, and uploading information to a database; supervision of local quality assurance by the PID physicians and hospital epidemiologists; and review and correction of reported data. During the monthly online meetings, the team reported the total patient days, the percentage of IPA and rates of HAI per 1000 patient days, and the line listings of all infected patients and of all microorganisms isolated, including the sources of the infections and the susceptibilities of the pathogens isolated. These monthly infection rate reports contributed to establishing expected HAI rates (28) for the SOLCA–Quito and UNOP POUs. The annual HAIs rates, calculated from the average monthly HAI rates, were used to establish the trends. The results (annual rates and trends) were presented to the hospital staff and leadership of SOLCA–Quito and UNOP during scheduled meetings and were helpful for formulating IPC strategic plans.
- Hand hygiene practices in the POU were assessed by using the World Health Organization (WHO) Hand Hygiene Self-Assessment Framework (HHSAF) (29). At regular intervals, the POUs evaluated their hand hygiene performance by gathering information on each of the five WHO HHSAF components, and they reported the results during the monthly meetings.
- Vascular access practice improvement consisted of annotating the types of vascular access used in the POU, for example, long-term (tunneled) central venous catheters (CVCs), short-term CVCs (nontunneled), peripherally inserted central catheters (PICCs), midline catheters, and PIV catheters (PIVCs). Information collected for CVCs included the duration of usage (access) and, for PIVCs, the duration of placement. For CVCs and PIVCs, there was daily inspection of vascular insertion sites and notation of any complications (infections, occlusion, extravasations, dislodgments, or physical damage to the catheters). The presence of phlebitis in PIV inserted vascular access was graded (from 1 to 4) (30) and was reported as monthly rates (the number of phlebitis events per 100 catheter days). CVC-associated bloodstream infections were reported in accordance with the standard definition and reporting system (31). At both POUs, all healthcare providers were trained annually by the IP in best practices for placing and accessing vascular catheter devices, following standard published guidelines (26).
- Blood cultures were performed for all children with indications for this practice. At both hospitals, the indications were as follows: the patient had febrile neutropenia with or without a clinical focus; the patient was suspected to have a catheter-related infection; the patient had a serious illness requiring antibiotic administration; or the patient was persistently febrile but the initial blood culture was negative (in this case, cultures were performed every 3 days, or more frequently if the patient was unstable). Blood cultures were also performed as follow-up after a positive blood culture to evaluate the clearance of bacteremia, starting 24–48 hours after a laboratory report of an initial positive blood culture. Since 2009, the UNOP microbiology laboratories have had a BacT/ALERT 3D Microbial Identification System (bioMérieux, Inc., Durham, NC), which is an automated alert system for positive blood cultures, and they use VITEK 2 panels (bioMérieux France, Craponne, France) for identifying pathogens. Also, since 2015, UNOP has had policies and procedures in place for obtaining blood cultures that were agreed upon by the microbiology and infectious diseases service staff. At SOLCA–Quito, similar microbiology laboratory equipment became available in 2018, and the infection care and IPC team and the microbiology laboratory staff recently finalized the policies and procedures for blood cultures. At both hospitals, the microbiology laboratory staff inform clinical staff about positive blood cultures on a 24/7 basis. The total numbers of blood cultures and positive cultures, the contamination rates, and the types of pathogens isolated were reported monthly, along with the incidence of multidrug-resistance organisms.

### 3.4 Training the POU staff

At the time this report was prepared, the SOLCA–Quito POU staff had 20 nurses, four hematologist/oncologists, four pediatricians, and eight physicians-in-training (third-year pediatric residents and generalists), one pediatric surgeon, one pediatric infectious diseases specialist, and one general psychologist. UNOP currently has 306 nurses, 13 oncologists, 23 pediatricians, 12 oncology fellows, five ICU specialists, and two ICU fellows; ID/IPC is supported by three ID specialists, three nurses, and a pharmacist. At both hospitals, at the onset of the collaboration, the IPC teams conducted a hand hygiene needs assessment by using the WHO HHSAF. The available IPC policies and procedures were then reviewed and updated. These policies and procedures were for hand hygiene, vascular access, and prevention of HAIs (including VAP, catheter-related infections, and surgical site infections). Training was conducted by the IPC teams, using existing institutional policies and procedures. The IPC teams trained the hospital staff once a year. The staff were also continuously trained using the “just-in-time” technique (25). Every year, the IPC team celebrated World Hand Hygiene Day (32) as an important IPC promotional activity.

### 3.5 Infection surveillance and auditing of IPC practice indicators

During the collaboration, we recorded multiple process and outcome metrics, including those related to the rates of infections (HAIs, CLABSIs, and CAUTIs); the grading and rates of phlebitis; the rates of blood culture positivity and contamination; and the accuracy, completeness, and timeliness of the data collection and monthly reports. Compliance with hand hygiene and decreasing rates of HAIs were process and outcome indicators for quality of care in the POU.

Total hospital days, types and rates of HAIs and IPAs, vascular access days (with a CVC or PIVC) and complications, urinary catheter days and complications, and hand hygiene compliance rates were audited and reported monthly. All POU admissions were recorded by the pediatric resident of the unit on the Blue Sheet data collection form provided (see **Supplementary Material**). The completeness and accuracy of the data and their alignment with established definitions were monitored by the ID and oncology supervisory personnel at SOLCA–Quito and by the ID physicians at UNOP. The Blue Sheet for each patient was closed upon discharge, and the data were entered into a database and analyzed monthly to prepare the required reports. For infection rate reports, the calculations used total patient days and device days, and values were given relative to 1000 patient or device days.

The IPC teams audited hand hygiene and vascular access with the HHSAF (33). Although the HHSAF results were provided at least twice a year, the use and availability of hand hygiene antiseptic soaps and alcohol gel were audited and reported monthly. At UNOP, hand hygiene compliance depended partly on local cultural factors (34), and maintaining a high level of compliance required close attention by the IPC team. Related promotional events included participation in institutional and worldwide celebrations of hand hygiene (32). The IPC teams also audited vascular access. The entry sites and trajectories of PIVCs and PICCs were inspected daily by nurses, and the presence of local inflammation (phlebitis) was graded, using established definitions, on a scale from 1 to 4 (30). The rates of these complications were reported monthly. CVC access was also inspected, and any local complications were noted and reported monthly.

### 3.6 Impact on HAI rates

**Figure 1:**
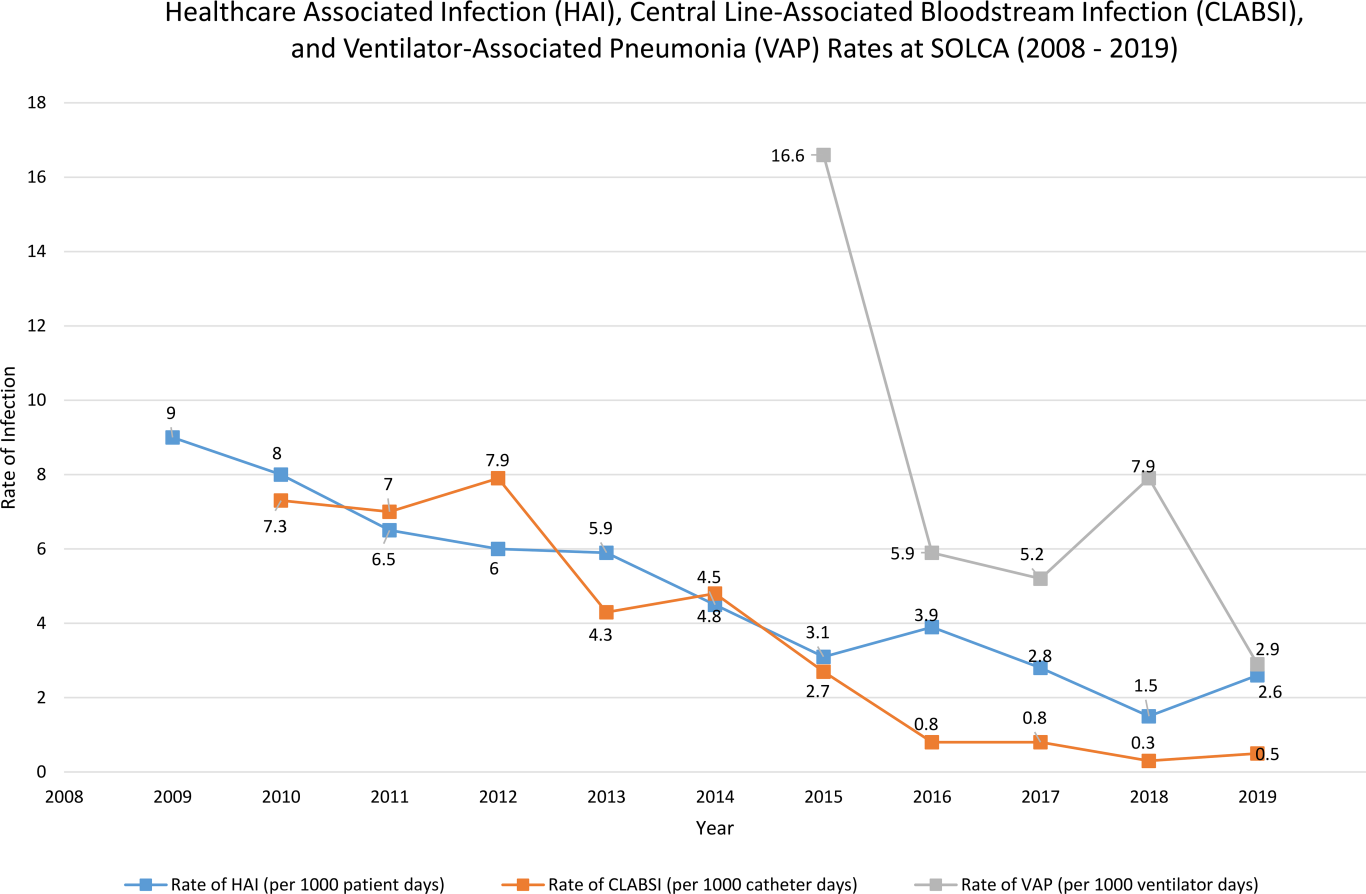
At Hospital SOLCA Quito between 2008 and 2019, healthcare-associated infection (HAI) rates decreased steadily (blue line). The central line-associated bloodstream infection (CLABSI) rate (orange line) fluctuated before dropping significantly by 2019. Ventilator-associated pneumonia (VAP) (gray line) saw the most dramatic change, with a sharp decline in 2016, leading to its lowest level by the end of the period.

**Figure 2:**
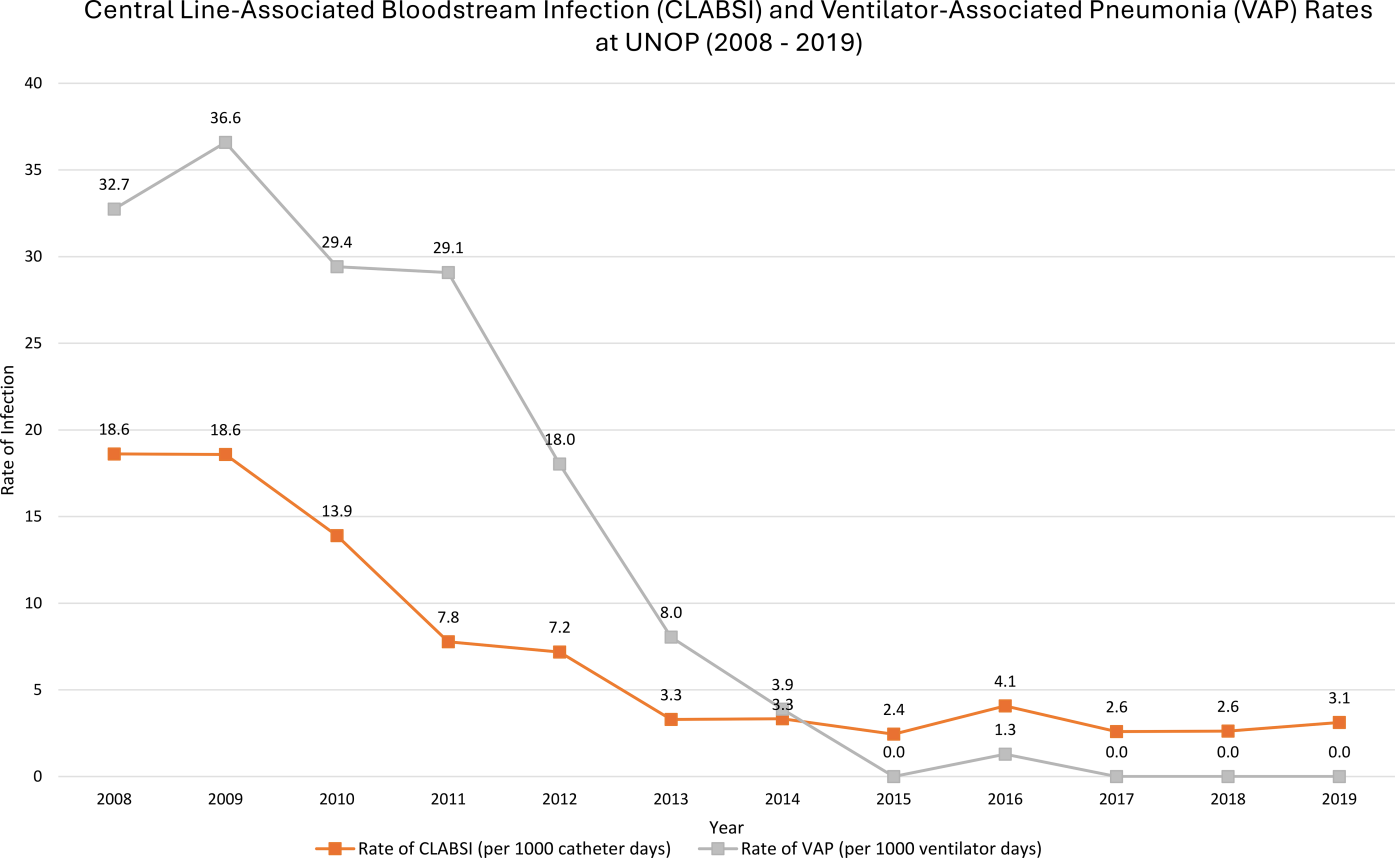
From 2008 to 2019, Hospital UNOP had significant success in reducing hospital-acquired infections. Rates of central line-associated bloodstream infections (CLABSI) (orange line) decreased substantially from 18.6 to 3.1, while ventilator-associated pneumonia (VAP) (gray line) rates dropped from an initial 36.6 to zero by 2015, and were sustained at zero through 2019.

Between the creation of the IPC team at Hospital SOLCA–Quito and the end of 2023, 16,284 children with cancer experienced a total of 80,789 hospital days and 333 HAI events. At baseline (2010), there were 9 HAIs/1000 patient days, whereas at the end of 2019, at the last assessment before the start of the COVID-19 pandemic, there were only 2.6 HAIs/1000 patient days (**Figure 1**). At UNOP, during the early years of IPC teamwork, there were 9.9 HAIs/1000 patient days in 2011, whereas in 2019 the rate decreased to 5.37 HAIs/1000 patient days. Post-pandemic, in 2023, the HAI rate was 8.69/1000 patient days. The CLABSI rate decreased from 32.75/1000 catheter days in 2008 to 3.11/1000 catheter days in 2019. Overall, the VAP rate decreased from 18.62/100 ventilator days in 2008 to 0 days in 2019 (**Figure 2**).

### 3.7 Expanding the program to other sites

To establish the teams at SOLCA–Quito and UNOP, we built a model based on the concept of an infection control liaison nurse or link nurse (35–37). The roles of an IPC liaison or link nurse are to participate in improving awareness and practice of IPC and conducting surveillance of HAIs in an assigned ward. In our model, which we called the “IPC link team,” the local pediatrician and the IP and support personnel assumed the key tasks of improving IPC practices through training staff, conducting surveillance of HAIs, and monitoring adherence to standard and transmission-based precautions in their POUs. During the development of the SOLCA–Quito and UNOP teams, and based on our growing experience and lessons learned in doing so, we began developing additional link teams at POUs at other sites. These POUs are in San Salvador, El Salvador; Tegucigalpa and San Pedro Sula, Honduras; Managua, Nicaragua; Tijuana, Mexico (38); and Davao, the Philippines.

More recently, the IPC link team model was adopted by the POUs of three hospitals on the island of Hispaniola. Importantly, the IPC practices of these link teams were disseminated throughout the host hospitals of the POUs and among members of the institutional IPC programs.

## 4 Discussion

An effective IPC program is necessary for a healthcare facility to provide a high-quality service, especially for patients who are immunosuppressed. Although the collaborations described in this report concentrated on the POUs, interventions such as improving the process for obtaining blood cultures, building the expertise of healthcare providers in placing vascular access, and providing training in infection care and IPC to pediatricians and nursing staff proved beneficial for the entire hospital.

Through collaborations, we built two IPC teams that resulted in the creation of an IPC link team in the POU of SOLCA–Quito and the strengthening of the IPC program at UNOP. In both cases, we started by establishing a strategic partnership between the St. Jude GIDP and the local hospital leaders with a commitment to support this effort. The initial planning and preparation were followed by building, developing, and sustaining the IPC teams through the years. The sustained institutional collaborations between the St. Jude GIDP and SOLCA–Quito and UNOP were guided by clear objectives and deliverables; they were supported by committed, resourceful, and culturally competent infection care and IPC experts; and they depended on excellent communication logistics. Identifying, training, and mentoring local champions with appropriate subject knowledge and cultural competence with respect to the local healthcare environment were essential for building effective IPC teams at these two hospitals. The trust of the local hospital leaders in the collaboration resulted in the eventual incorporation of the IPC teams and their functions into the institutional IPC structure.

A recent global survey conducted using the online WHO IPC Assessment Framework (IPCAF) obtained 4440 responses from 81 countries. In this survey, IPC programs in low-resource settings had significantly lower scores than those in HICs, and only 15.2% of facilities met all of the IPCAF minimum requirements (39). Furthermore, Magrath et al. (40) reported that although the global burden of cancer in children is low, the great majority of cancer cases, representing 84% of childhood cancers, occur in LMICs, where access to care is poor. In a study of general hospitals in the United States, the characteristics of effective IPC programs were organized surveillance and control of infections, the presence of a trained IP, and the existence of a system for reporting infection rates to healthcare providers. Hospitals with this type of program had an HAI rate 32% lower than that of hospitals without such a program (41). Implementing a high-quality and effective IPC program with trained team members is key to promoting safety at pediatric oncology institutions, especially those in LMICs.

A key step in the collaborations was identifying, training, and mentoring a local champion in infection care and IPC to aid in attaining our goals. Guidelines published by international health agencies, including the WHO (42), the CDC (43), the National Health Service (NHS) of the United Kingdom (44), and Infection Prevention and Control Canada (IPAC Canada) (45), offer guidance on improving IPC capacity. In the WHO guidelines on core components of IPC programs (27), one requirement is the presence of a dedicated and trained preventionist; the CDC infection control website (43) provides educational materials for improving knowledge and competencies of preventionists; the NHS (44) has outlined a framework for training IPs and hospital staff in IPC; and the IPAC Canada website (45) displays the core competencies for IPs. The COVID pandemic inflicted a heavy burden on IPC personnel, but it also highlighted the importance of IPC.

Organizations such as Ascension, a non-profit healthcare system in the United States, supported their IPs during this health emergency by empowering them and establishing partnerships through coaching and consultation with more experienced IPs, providing access to training and certifications, facilitating associations with other key stakeholders, and promoting the use of standardize IPC tools (46). When we initiated our training program, we relied on both existing and newly created training resources (24, 47). Importantly, for the duration of the collaboration, we trained, coached, mentored, and partnered with our IPs in quality improvement and research projects (48). Local IPC champions are important contributors to knowledge dissemination and promotion, and they are leaders in infection prevention (15). We found that training and equipping our local champions was essential to building an effective IPC team.

For surveillance of infections, the IPC teams at SOLCA–Quito and UNOP reviewed and used the Blue Sheet patient data collection forms provided and populated an Epi Info database with the information collected. This information enabled numerator and denominator values to be calculated for hospitalized infected and non-infected children, and analysis of the cumulative monthly data enabled reporting of the infection rates and the role of risk factors during hospitalization. In the seminal work of Haley et al. (41), the presence of surveillance and control programs in the hospitals studied was associated with there being fewer HAIs than in hospitals lacking such activities. In the WHO IPC guidelines (42), surveillance is one of the eight core components of effective IPC programs. The guideline states that HAI surveillance can inform IPC strategies but that the quality and utility of the data will depend on the use of standard definitions tailored to the needs of the institution. The CDC also includes surveillance and reporting of infections among the minimal standards for safety of care in outpatient settings (49). Surveillance data from the POUs in Ecuador and Guatemala were indicators of healthcare quality performance and guided the improvement efforts of the IPC teams.

The Blue Sheet consisted of a comprehensive list of clinical variables that a hospitalized child could experience and the impact of the types, rates, and outcomes of infection. This data collection form used the standard CDC HAI definitions (50), and instructions were provided on how to complete the form and how to populate the Epi Info database with the information collected. The monthly reports produced by the IPC teams eventually enabled them to establish their own infection rate thresholds, to observe trends, to make comparisons with other institutions, and to use the information in making decisions regarding IPC interventions or in communications with interested local stakeholders. HAI rates are affected by multiple factors, including those related to the patient, the healthcare providers, and the environment. COVID-19 had a profound impact on UNOP; for example, the HAI rates increased from 5.37 in 2019 to 8.69 in 2023. This increase was primarily due to nosocomial transmission of respiratory viruses, facilitated by increased patient load, reduced hospital budgets, staff attrition, decreased hygiene compliance, and modification of hospital processes to incorporate COVID-19 precautions. Since then, the UNOP plans have included improved IPC budgeting, reduced staff attrition, and improved education and monitoring of IPC precautions, including triage of sick staff.

Periodic monitoring of the data collection and review of the definitions used gradually improved the IPC team surveillance and IPC auditing performance. For the surveillance data to be actionable within the institution, they must be of good quality. To report reliable outcomes and process metrics, the data quality is critical. Data quality can be improved by standardizing operational procedures, by training personnel in using data collection tools, by providing timely feedback based on analyzed data to involved and interested individuals, and by assuring clarity in the use of denominators and numerators in calculations (51). Our IPC project introduced monthly reporting of healthcare quality indicators, such as rates of HAIs and hand hygiene compliance, that were not previously reported. Beneficial responses to surveillance data should focus on interpreting the data and performing interventions if necessary. For example, through surveillance, French et al. learned that hydrogen peroxide vapor in terminal decontamination cleaning was effective in controlling an MRSA outbreak (52). At SOLCA–Quito and UNOP, the IPC teams and the recipients of the reports gradually became accustomed to the surveillance information. They used it to identify areas of high infection and poor IPC practice compliance and to provide a rationale for implementing strategies and requesting resources to reduce the occurrence of poor-quality indicators. Since the start of the collaboration in 2009, in the POUs at SOLCA–Quito and UNOP, the active surveillance program has resulted in HAI-related indicators being consistently reported and used as quality indicators. By using surveillance, these two POUs were able to report HAI rates consistently and to establish goals for IPC interventions.

Throughout our collaboration, multiple actions within the framework of our model were led by the IPC team, with coaching by the St. Jude GIDP partners. Although the “whats” were clear from multiple guidance sources, the “hows” were more vague and sparser. In the early 2000s, the WHO initiated efforts to reduce HAIs through its Clean Care is Safer Care initiative. This initiative subsequently developed into a comprehensive guideline (42) that addressed the eight core components that are deemed essential to an institutional IPC program. The Joint Commission, an agency that reviews and accredits the safety and quality of services of healthcare institutions in the United States, offers on its latest website an extensive collection of toolkits, standards, and evidence-based information for IPC practice (53). More recently, the CDC has published a collection of guidance on IPC core practices, training, and tools (43). The SOLCA–Quito and UNOP IPC team collaborations used multiple elements of these guidelines in building the team, training the personnel, implementing surveillance, and monitoring IPC practices; however, coaching and mentoring (“hows”) were essential to keep the team focused and engaged.

At the time of our initial engagement with SOLCA–Quito and UNOP, building an IPC team and incorporating its function into the institutional structure represented an innovation process for the quality of care. Rogers defines innovation as “an idea, practice, or object that is perceived as new by an individual or other unit of adoption” (54), and our implementation of IPC teams followed elements of the diffusion and adoption of an innovation (54). Predictors for adoption are existing beliefs regarding the value and benefits of the innovation and its compatibility with the implementing ecosystem (55). Facilitators of the innovation adoption at SOLCA–Quito and UNOP were the launch by the WHO of a hand hygiene campaign around the time that the IPC teams were initiated, frequent presentations on the problem of HAIs in pediatric cancer and how IPC could help in decreasing their incidence, and the support of the work of the IPC team by local leaders. Throughout the collaboration, the GIDP and the local teams from SOLCA–Quito and UNOP maintained close communication with both institutions in Ecuador and Guatemala, and M.M. and J.J.A. became integral members of the hospital quality offices. The data (rates of HAI and IPC processes) generated by the IPC Link Teams, previously nonexistent, provided objective information on the quality of healthcare. Notably, the financial savings that resulted from avoiding additional expenses associated with HAI testing, antibiotic use, and increased hospitalizations were crucial to management acceptance of the IPC program. Management subsequently allocated a budget for IPC supplies and supported training for all healthcare staff to ensure that they used similar language. Ultimately, the most important factor for sustainability was maintaining close communication with hospital management and demonstrating the usefulness and financial benefits of IPC. Importantly, 6 years after the initial funding period was concluded, the SOLCA–Quito IPC team continues to perform as link team to the pediatric oncology ward and collaborates with the institutional IPC program by providing surveillance data and assisting in staff IPC training. Similarly, the UNOP IPC team members became the institutional IPC program staff, and they also continue to conduct surveillance of infections, monitor IPC practices, and engage in staff IPC training. Therefore, the project has proved to be sustainable at both institutions (56).

There are several limitations to this report, including the retrospective nature of our data review and the fact that we are reporting events at two different institutions, albeit ones treating similar types of patients. We have tested the utility of our methods for implementing IPC teams in other POUs, such as ones in Mexico (38) and Pakistan (57), and have achieved similar success. Institutions in several countries in Central America (El Salvador, Honduras, and Nicaragua) and in the Caribbean (Haiti and the Dominican Republic) have used the same methodology to improve IPC in their POUs.

### 4.1 Conclusions

We successfully decreased HAIs in two POUs through a collaboration partnership, and we sustained an IPC program and an IPC link team at two institutions. In LMICs, deficiencies in hospital-based IPC programs and high rates of HAIs are reported especially among complex patients (cancer, transplants, cardiovascular surgeries, intensive care units), regardless of age (4). While an effective IPC program for these services is expensive and the supporting budget may be prohibitive for hospitals in LMICs, investing in an IPC link team, such as in our model, could be a solution to compensate for the shortcomings of a traditional IPC program. IPC link teams could be established for units that require closer monitoring of HAIs, optimization of antimicrobial use, staff education and training, robust policies and procedures, and monitoring of compliance with these procedures. However, IPC link team members should receive additional training to ensure optimal IPC competencies, as they will be responsible for raising awareness of infection control practices, promoting policy compliance, and facilitating communication between the infection control team and frontline healthcare professionals (58).

Although the HAI rates at SOLCA–Quito and UNOP were higher than those in HICs, the rates were lower than those reported in LMICs (4). This model has been successfully used at multiple sites, demonstrating its utility and reproducibility mainly, but perhaps not exclusively, in POUs. Several of the WHO recommendations that we implemented when building these structures were subsequently assimilated by the institutions, thereby adding an important element of quality of care for children with cancer, encouraging better IPC practices, and—it is hoped—improving survival. The IPC link team at SOLCA–Quito continues to perform well, whereas at UNOP, the IPC team members and their functions were ultimately integrated into the institutional structure, becoming an essential component of the facility. A link team is an excellent model for improving the quality of care for children with cancer, a population at high risk for infection acquisition and transmission, especially at large institutions with suboptimal IPC resources that cannot meet the needs of the POUs, as is often the case at hospitals in low-resource settings.

## Data Availability

The raw data supporting the conclusions of this article will be made available by the authors, without undue reservation.
