# Supplementary material for: Building and sustaining infection prevention and control teams at two pediatric cancer units in Ecuador and Guatemala through a collaboration partnership
Source: Front Oncol. 2025 Sep 25;15:1577066. doi: 10.3389/fonc.2025.1577066 (PMC12507594; doi:10.3389/fonc.2025.1577066)
Supplement: Supplementary file 1 [file DataSheet1.pdf]

Registry of Infections and their Risk Factors

Important: Fill out this form for each patient and each admission; it is critical to write the date in day/month/year format

Date ___/___/____ (day/month/year) Unit______________________________ Patient Number: ______________ ID or POND #_____________

A. Patient Demographics — Comments

Name: ____ ______________________

Date of Birth ___/___/____ (day/month/year) Age _________ (years) Sex ☐ M ☐ F ______________________

Date of Admission ___/___/____ Date of Discharge ___/___/____ ______________________

Admission Dx ______________________________ Oncologic Dx________________________________ ______________________

Admitted to ICU ☐ Yes ☐ No Duration (days) ________ HAI[1] acquired in ICU? ☐ Yes ☐ No ______________________

B. Exposure to Risk Factors — Comments

1. Mechanical Ventilation ☐ Yes ☐ No Duration (days) __________ ______________________
2. Vascular Catheters
   - Peripheral (Vein) #_____ Days______ Peripheral (Artery) #_____ Days______ ______________________
   - Central Non-Tunneled #_____ Days______ Central Tunneled #_____ Days______ ______________________
   - PICC #_____ Days______ Totally Implantable #_____ Days______ ______________________
4. Urinary Catheter ☐ Yes ☐ No Duration (days) ______ ______________________
5. Surgery Date ___/___/___ Principal Procedure________________ Duration: _______ ______________________
   - Type of wound: ☐ Clean ☐ Clean Contaminated ☐ Contaminated ☐ Dirty Infected ASA Code: ____ ______________________
6. Neutropenia ☐ < 500 NT Duration (days): ________ ☐ ≤ 100 NT Duration (days): _______ ______________________
7. Chemotherapy: ☐ Yes ☐ No Protocol ______________ Date of last dose of chemo ___/___/_____ ______________________
   - Phase of treatment: ☐ Induction ☐ Consolidation ☐ Maintenance ☐ Relapse ☐ Does not apply ______________________
8. Other: ______________________ Date ___/___/___ ______________________________ ______________________

C. Infections

C. Infections (see definitions)	Date of onset (day/month/year)	HAI[1]		Cultures[2]			Prev HAI[3]	DaysPre HAI[4]	Microorganism / Comments [5] HAI acquired in[6]
1. CNS									
☐ Intracranial	___/___/____	☐ Yes	☐ No	☐ Pos	☐ Neg	☐ N/D	☐ Yes	____	______________________
☐ Meningitis or Ventriculitis	___/___/____	☐ Yes	☐ No	☐ Pos	☐ Neg	☐ N/D	☐ Yes	____	______________________
2. SKIN AND SOFT TISSUE									
☐ Phlebitis	___/___/____	☐ Yes	☐ No	☐ Pos	☐ Neg	☐ N/D	☐ Yes	____	______________________
Grade: #____ I #____ II #____ III #____ IV									
☐ Skin Infection	___/___/____	☐ Yes	☐ No	☐ Pos	☐ Neg	☐ N/D	☐ Yes	____	______________________
☐ Soft Tissue Infection	___/___/____	☐ Yes	☐ No	☐ Pos	☐ Neg	☐ N/D	☐ Yes	____	______________________
☐ Scabies	___/___/____	☐ Yes	☐ No	☐ Pos	☐ Neg	☐ N/D	☐ Yes	____	______________________
3. SURGICAL SITE INFECTION									
☐ Superficial ☐ Deep ☐ Organ/Space	___/___/____	☐ Yes	☐ No	☐ Pos	☐ Neg	☐ N/D	☐ Yes	____	______________________
4. RESPIRATORY INFECTION									
☐ Upper Respiratory	___/___/____	☐ Yes	☐ No	☐ Pos	☐ Neg	☐ N/D	☐ Yes	____	______________________
☐ Pneumonia	___/___/____	☐ Yes	☐ No	☐ Pos	☐ Neg	☐ N/D	☐ Yes	____	______________________
☐ Clinical ☐ Pneumonia with uncommon pathogens ☐ Pneumonia with common pathogens ☐ In an immunocompromised patient									
☐ Lower Respiratory	___/___/____	☐ Yes	☐ No	☐ Pos	☐ Neg	☐ N/D	☐ Yes	____	______________________
5. EYE, EAR, NOSE, THROAT and MOUTH									
☐ Conjunctivitis ☐ Eye (other) ☐ Otitis	___/___/____	☐ Yes	☐ No	☐ Pos	☐ Neg	☐ N/D	☐ Yes	____	______________________
☐ Oral Cavity Infection	___/___/____	☐ Yes	☐ No	☐ Pos	☐ Neg	☐ N/D	☐ Yes	____	______________________
Type: ☐ Bacterial ☐ Viral ☐ Fungal ☐ Mucositis Grade: #____ I #____ II #____ III #____ IV									
☐ Mastoiditis ☐ Sinusitis	___/___/____	☐ Yes	☐ No	☐ Pos	☐ Neg	☐ N/D	☐ Yes	____	______________________
6. GASTROINTESTINAL									
☐ Gastroenteritis	___/___/____	☐ Yes	☐ No	☐ Pos	☐ Neg	☐ N/D	☐ Yes	____	______________________
☐ Esophagus, Stomach, Small and Large Bowel, Rectum	___/___/____	☐ Yes	☐ No	☐ Pos	☐ Neg	☐ N/D	☐ Yes	____	______________________
☐Hepatitis ☐ Intraabdominal	___/___/____	☐ Yes	☐ No	☐ Pos	☐ Neg	☐ N/D	☐ Yes	____	______________________
☐ Other: ______________	___/___/____	☐ Yes	☐ No	☐ Pos	☐ Neg	☐ N/D	☐ Yes	____	______________________
7. GENITOURINARY									
☐ Symptomatic	___/___/____	☐ Yes	☐ No	☐ Pos	☐ Neg	☐ N/D	☐ Yes	____	______________________
☐ Asymptomatic	___/___/____	☐ Yes	☐ No	☐ Pos	☐ Neg	☐ N/D	☐ Yes	____	______________________
☐ Other Urinary Tract Infections	___/___/____	☐ Yes	☐ No	☐ Pos	☐ Neg	☐ N/D	☐ Yes	____	______________________

[1]HAI = Healthcare Associated Infection, [2]Abreviations used: Pos = Positive, Neg = Negative, N/D = Not Done

[3] Was this infection an HAI from a previous admission? [4]DaysPreHAI = Days from admission to the development of HAI

[5] Number the microorganisms in the order that the infections appear. This order should be maintained in Part E.

[6] If the infection was healthcare associated, write the unit in which it was acquired in this column.

Registry of Infections and their Risk Factors

Important: Fill out this form for each patient and each admission; it is critical to write the date in day/month/year format

C. Infections (see definitions)	Date of onset *(day/month/year)*	HAI[1]		Cultures[2]			Prev HAI	DaysPre HAI[3]	Microorganism / Comments [4] HAI acquired in[5]
8. BLOODSTREAM									
☐ Bacteremia ☐ Clinical Sepsis	__/__/____	☐ Yes	☐ No	☐ Pos	☐ Neg	☐ N/D	☐ Yes	____	_______________________
9. CARDIOVASCULAR									
☐ Arterial or Venous	__/__/____	☐ Yes	☐ No	☐ Pos	☐ Neg	☐ N/D	☐ Yes	____	_______________________
10. SYSTEMIC									
☐ Varicella Zoster	__/__/____	☐ Yes	☐ No	☐ Pos	☐ Neg	☐ N/D	☐ Yes	____	_______________________
☐ Disseminated Herpes	__/__/____	☐ Yes	☐ No	☐ Pos	☐ Neg	☐ N/D	☐ Yes	____	_______________________
11. Other__________________	__/__/____	☐ Yes	☐ No	☐ Pos	☐ Neg	☐ N/D	☐ Yes	____	_______________________
12. Other__________________	__/__/____	☐ Yes	☐ No	☐ Pos	☐ Neg	☐ N/D	☐ Yes	____	_______________________
13. Infection(s) reported coincide with neutropenia[6] (<500)				☐ Yes	☐ No				_______________________

D. Antimicrobials

Treatment with antimicrobials: Use the abbreviations written in the table below. If the antimicrobial does not appear in the table, write out the full name of the antimicrobial.

Antimicrobial	Start Date	End Date	Prophylaxis	Preemptive	Therapeutic	Comments
________________	__/__/____	__/__/____	☐	☐	☐	__________________
________________	__/__/____	__/__/____	☐	☐	☐	__________________
________________	__/__/____	__/__/____	☐	☐	☐	__________________
________________	__/__/____	__/__/____	☐	☐	☐	__________________
________________	__/__/____	__/__/____	☐	☐	☐	__________________
________________	__/__/____	__/__/____	☐	☐	☐	__________________
________________	__/__/____	__/__/____	☐	☐	☐	__________________
________________	__/__/____	__/__/____	☐	☐	☐	__________________

Continue on another page if needed

E. Microorganisms y Susceptibilities[7,8]

Microorganism	AMK	GEN	CTX	CRO	CAZ	CIP	CLI	IPM	MER	OXA	PEN	AMP	TAZ	PIP	VAN	Fluc			
1																			
2																			
3																			
4																			
5																			
6																			
7																			

[6] Write the corresponding numbers in the comments section

[7] Write the number of each organism from Part E to the corresponding infection in Part C.

Table of Abbreviations of Antimicrobials

Acyc	Acyclovir	CAZ	Ceftazidime	LVX	Levofloxacin	PIP	Piperacillin
AMK	Amikacin	CRO	Ceftriaxone	LNZ	Linezolid	Posa	Posaconazole
AMC	Amoxicillin	CXM	Cefuroxime	MER	Meropenem	RIF	Rifampin
Amph	Amphotericin	CHL	Chloramphenicol	MEZ	Mezlocillin	SSS	Sulfonamides
AMP	Ampicillin	CIP	Ciprofloxacin	MNO	Minocycline	TAZ	Tazobactam
ATM	Aztreonam	CLI	Clindamycin	NIT	Nitrofurantoin	TEC	Teicoplanin
CRB	Carbenicillin	DOX	Doxycycline	NOR	Norfloxacin	TCY	Tetracycline
Casp	Caspofungin	ERY	Erythromycin	NOV	Novobiocin	TIC	Ticarcillin
CEP	Cefalotin	Fluc	Fluconazole	OFX	Ofloxacin	TOB	Tobramycin
MAN	Cefamandol	Gang	Gangcyclovir	Osel	Oseltamivir	STX	Trimethoprim/ Sulfametoxazol
CTX	Cefotaxime	GEN	Gentamicin	PEN	Penicillin	Vori	Voriconazole
FOX	Cefoxitin	IPM	Imipenem				

[8] Use abbreviations:

Sensitive – S
Intermediate - I
Resistant – R
Not Done - ND

Name (Data Collection):____; __/__/____ (Date data collected)
Name (Supervisor): ____;__/__/____ (Date approved)
Name (Data Entry): ____;__/__/____ (Date data entry was completed)
